# Antimicrobial Properties Related to Anti-Acne and Deodorant Efficacy of *Hedychium coronarium* J. Koenig Extracts from Pulsed Electric Field Extraction

**DOI:** 10.3390/antibiotics13010108

**Published:** 2024-01-22

**Authors:** Manasanan Mitchaleaw, Saranya Juntrapirom, Anurak Bunrod, Watchara Kanjanakawinkul, Artit Yawootti, Wannaree Charoensup, Sasithorn Sirilun, Wantida Chaiyana

**Affiliations:** 1 Department of Pharmaceutical Sciences, Faculty of Pharmacy, Chiang Mai University, Chiang Mai 50200, Thailand; manasanan_mit@cmu.ac.th (M.M.); wannaree.charoensup@cmu.ac.th (W.C.); sasithorn.s@cmu.ac.th (S.S.); 2 Chulabhorn Royal Pharmaceutical Manufacturing Facilities by Chulabhorn Royal Academy, Chon Buri 20180, Thailand; saranya.jun@cra.ac.th (S.J.); anurak.bun@cra.ac.th (A.B.); watchara.kan@cra.ac.th (W.K.); 3 Department of Electrical Engineering, Faculty of Engineering, Rajamangala University of Technology Lanna, Chiang Mai 50300, Thailand; yartit@rmutl.ac.th; 4 Center of Excellence in Pharmaceutical Nanotechnology, Faculty of Pharmacy, Chiang Mai University, Chiang Mai 50200, Thailand

**Keywords:** *Hedychium coronarium*, pulsed electric field, antimicrobial activities, acne, deodorant

## Abstract

This study investigated the potential of pulsed electric field (PEF) extraction in enhancing the antimicrobial properties related to anti-acne and deodorant properties of *Hedychium coronarium* extract. The dried leaf and rhizome of *H. coronarium* were extracted using 95% *v*/*v* ethanol through both conventional solvent extraction and PEF extraction techniques (10, 14, and 20 kV/cm). The chemical composition of the extracts was analyzed. The antimicrobial activities, specifically in relation to acne treatment against *Cutibacterium acnes* and deodorant properties against *Staphylococcus aureus*, *Bacillus subtilis*, *Micrococcus luteus*, *Pseudomonas aeruginosa*, and *Escherichia coli*, were determined. The irritation profile of was evaluated using the hen’s egg chorioallantoic membrane test. The results showed that PEF extraction increased the extract yield, particularly at an electric field strength of 20 kV/cm. Furthermore, PEF extraction significantly enhanced the ellagic acid content, particularly in the leaf extract. Furthermore, the leaf extract demonstrated stronger inhibitory effects against microorganisms associated with body odor and acne compared to the rhizome extract. Notably, all extracts exhibited no signs of irritation, indicating their safety. Overall, the findings suggest that PEF extraction from *H. coronarium* enhances yield, bioactive compound content, and antimicrobial effects. This indicates the potential of the extract for acne treatment and deodorant use.

## 1. Introduction

*Hedychium coronarium* J. Koenig, commonly known as butterfly ginger and belonging to the Zingiberaceae family, is highly regarded for its vibrant and fragrant flowers, and it is widely distributed in tropical and subtropical regions across the globe [1]. This species is known by various common names such as butterfly lily, cinnamon jasmine, garland flower, and ginger lily, reflecting its ornamental beauty and versatile applications in traditional medicine [2]. *H. coronarium* has been cultivated as a crop in certain regions since it is primarily valued for its ornamental beauty [3]. The alluring fragrance of *H. coronarium* flowers, along with the presence of aromatic compounds that enhance their overall appeal with a distinctive and pleasing note, has established them as a favored option for perfume production. These flowers are especially fragrant during the morning and evening hours until dusk [4]. Furthermore, the inherent resistance of *H. coronarium* flowers to insects enables them to bloom gradually while preserving their fragrance over time [5]. Cultivating *H. coronarium* for flower harvesting in the perfume industry often generates a substantial amount of agricultural waste due to the practice of uprooting and replanting, which is typical for plants in the Zingiberaceae family. In this process, the leaves and rhizomes of *H. coronarium* are considered as by-products. Indeed, the by-products of *H. coronarium*, including the leaves and rhizomes, have the potential for beneficial utilization in various domains. Their utilization would enhance their value, minimize waste, and contribute to sustainability and resource optimization.

The rhizome and leaf of *H. coronarium* exhibit significant antimicrobial activity, making them valuable for various applications [6]. Essential oils from both leaf and rhizome show remarkable antimicrobial activity against a range of fungal and bacterial strains [7]. Previous studies reported that the rhizome essential oil demonstrates antimicrobial properties against bacteria, such as *Salmonella Typhi*, *Escherichia coli*, and *Proteus vulgaris*, as well as fungi, including *Candida albicans* and *Candida glabrata* [8]. Furthermore, the methanol and dichloromethane extracts from the rhizome display antibacterial activity against both Gram-positive and Gram-negative bacteria [9]. These findings highlight the potential of *H. coronarium*’s rhizome and leaf as sources of antimicrobial compounds, which can have applications in pharmaceutical, agricultural, and cosmetic industries.

The significant applications of natural extracts with antimicrobial properties include anti-acne and body odor reduction. This is supported by a sizable and rising market for these kinds of products, which is currently being driven by an established consumer preference for natural extracts [10]. *Cutibacterium acnes* (previously known as *Propionibacterium acnes*), which is important in the control of skin homeostasis and inhibits colonization by other harmful microbes, can also act as an opportunistic pathogen in acne vulgaris by promoting sebum accumulation in the infundibulum and inducing keratinocyte proliferation, resulting in pilosebaceous unit blockage, and eventually triggering an inflammatory response within the pilosebaceous unit [11,12]. Therefore, compounds with an antimicrobial effect against *C. acnes* have been widely used for acne treatment. However, the use of antibiotics led to the induction of antibiotic resistance; for example, up to 40% of *C. acnes* strains have been found to be resistant to erythromycin, clindamycin, and tetracycline, resulting in an increased likelihood of treatment failure [12]. Natural compounds have hence received overwhelming attention. Aside from acne, which is not a serious disease but can have serious consequences both physically and psychologically, bad body odor can influence how individuals assess others and subsequently cause them to adjust their behavior accordingly [13]. Body odor is a distinctive human attribute caused by the bacterial breakdown of odorless natural secretions into volatile odorous chemicals [14]. The sweat component metabolized by *Staphylococcus aureus* results in diacetyl, which imparts an acid-like quality to axillary and foot odor [15]. On the other hand, *Bacillus subtilis* has been detected in the plantar skin of subjects with strong foot odor [16]. *Micrococcus luteus* has been reported to be among the Gram-positive bacteria in the mixture of bacterial flora of the axilla that cause armpit malodor [17]. Furthermore, *Pseudomonas aeruginosa* and *E. coli* have also been reported as the causes of body odor [18,19]. Therefore, inhibition of these microbes would be one strategy to prevent unpleasant body odor.

While essential oils have demonstrated their effectiveness, it is important to acknowledge potential drawbacks when considering their topical applications, particularly in relation to skin irritations. On the other hand, the extraction process using organic solvents can potentially raise environmental concerns [20]. Therefore, the concept of “green extraction” has emerged as an intriguing approach. Green extraction refers to the use of sustainable and environmentally friendly techniques to extract valuable compounds from plant materials [21]. Furthermore, the choice of solvent used in various extraction processes is another crucial aspect to consider in terms of sustainability and environmental impact. Ethanol is often considered a greener solvent compared to many other organic solvents since it is derived from renewable resources such as plant biomass, and its use can reduce environmental impacts when compared to petroleum-based solvents [22]. Ethanol is biodegradable, has low toxicity, and is widely used in various industries, including extraction processes [23]. Although the antimicrobial activities of *H. coronarium* have been previously reported, most of the existing research focused on its essential oils. Only a few studies reported the antimicrobial effects of *H. coronarium* extracts. Das and Nayak (2022) reported that ethanolic extracts of *H. coronarium* could inhibit Gram-positive bacteria (*S. aureus*) and Gram-negative bacteria (*E. coli*) [3]. However, the extraction process was conventional maceration, employing heat treatment at 70 °C for 4–8 h [3]. The current study introduced a novel approach by utilizing a green extraction solvent alongside a pulse electric field (PEF) method to extract bioactive compounds from *H. coronarium.*

PEF is considered a green extraction technique. PEF involves the application of short, high-voltage pulses to the plant material, which creates pores in the cell membranes and facilitates the release of intracellular compounds [24]. This non-thermal extraction method offers several advantages from a sustainability perspective. PEF extraction operates at ambient or mild temperatures, eliminating the need for high heat, which could lead to degradation or loss of heat-sensitive compounds [25]. This reduces energy consumption and preserves the biologically active component and functional properties of the extracts.

Therefore, this study aimed to investigate the impact of the PEF technique in combination with ethanol as the extraction solvent to obtain bioactive compounds from the leaf and rhizome of *H. coronarium.* Furthermore, the antimicrobial activities for potential application in acne treatment and deodorant products, as well as the irritation potential of each extract, were also evaluated.

## 2. Results and Discussion

### 2.1. H. coronarium Extracts

**Figure 1:**
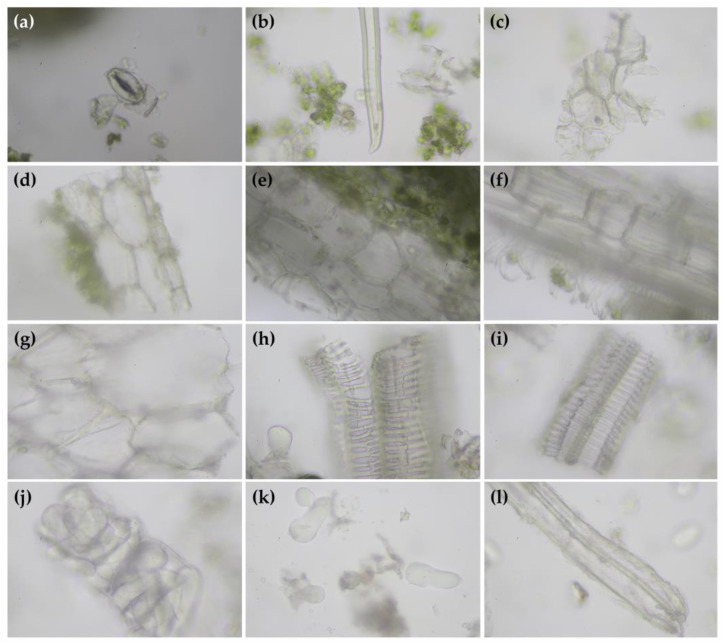
Microscopic images of *H. coronarium* leaf powder identified as isolated stomata (**a**), unicellular covering trichome (**b**), epidermis (**c**), epidermis with hypodermis below in sectional view (**d**), parenchyma (**e**), and vascular buddle beneath the parenchyma showing spiral vessels and fibers (**f**). Microscopic images of *H. coronarium* rhizome powder identified as parenchyma (**g**), reticulate (**h**), sclariform vessel (**i**), parenchyma filled with starch granules (**j**), starch granules and oil droplets (**k**), and fiber (**l**).

The microscopic structures of dried powder of *H. coronarium* leaves and rhizomes are shown in Figure 1. Parenchyma, characterized as thin-walled cells that remain capable of cell division even when mature [26], was found in both leaf and rhizome. The parenchyma showed spiral vessels and fibers in the leaf part, whereas the parenchyma was filled with starch granules in the rhizome part. On the other hand, stomata, trichome, epidermis, and vascular buddle were found in the *H. coronarium* leaf, while reticulate, sclariform vessels, starch granules, oil droplets, and fiber were found in the *H. coronarium* rhizome.

The yields of extracts from *H. coronarium* leaf and rhizome, based on dried plant weight, are shown in Table 1. Comparing various *H. coronarium* parts, there was no significant difference in the yields of the leaf and rhizome extracted from maceration and PEF extraction with the treatment of 20 kV/cm. However, at low intensities of PEF treatment (10 and 14 kV/cm), leaves yielded significantly higher extract contents (*p* < 0.05). The likely explanation could be the higher content of hydrophilic compounds in the leaf compared to the rhizome, which was consistent with a previous study that reported that the essential oil from the leaves, which was a combination of lipophilic compounds, was found to be less than that found in the rhizome (the yields were 0.15 and 0.20%, respectively) [7]. The findings noted that a distinct difference was observed when extracted using a shorter duration or with lower PEF intensities. This could be due to the fact that the mechanism of extracting the bioactive compounds from plant cells is diffusion of the compounds from a higher concentration (in the plant cell) to a lower concentration (in the extracting solvent). No significant difference could be observed in the extraction of maceration (using a longer duration of 3 cycles × 24 h) and PEF extraction using high intensity (20 kV/cm) due to the extraction reaching its steady state.

On the other hand, it was discovered that increasing the strength of PEF in both rhizome and leaf extracts resulted in greater extract yields. This phenomenon can be attributed to the increased efficiency in breaking down the cell walls, thereby facilitating the extraction of bioactive compounds from within the plant cells [27]. The PEF intensity of 20 kV/cm was suggested since it could yield a comparable content of the extracts. In addition to the comparable yield of the *H. coronarium* extracts, PEF contributed to shortening the extraction time. Furthermore, one of the advantages of PEF extraction is the significant reduction in solvent usage. With just a single 5 min cycle, PEF extraction yielded higher extract content compared to the traditional maceration method requiring three 24 h cycles of extraction. This demonstrated the efficiency and effectiveness of PEF in extracting desired compounds while minimizing solvent consumption [28]. In addition, PEF extraction has been widely scaled up to an industrial level [29,30] and can play a crucial role in sustainable processing systems by enhancing energy efficiency and requiring fewer resources to produce the extracts [31].

### 2.2. Chemical Compositions of H. coronarium Extracts

**Figure 2:**
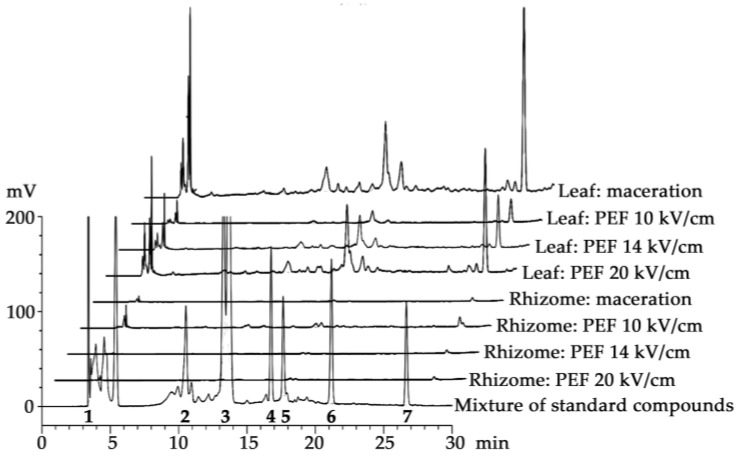
HPLC chromatograms of the extracts from *H. coronarium* leaf and rhizome from conventional maceration and PEF extractions using various PEF intensities of 10, 14, and 20 kV/cm in a comparison with the mixture of standard compounds, including kojic acid (**1**), chlorogenic acid (**2**), epigallocatechin gallate (EGCG) (**3**), rutin (**4**), ellagic acid (**5**), rosmarinic acid (**6**), and quercetin (**7**). PEF = pulse electric field extraction.

HPLC chromatograms of *H. coronarium* extracts are presented in Figure 2, along with a comparison to standard compounds, including kojic acid, chlorogenic acid, epigallocatechin gallate (EGCG), rutin, ellagic acid, rosmarinic acid, and quercetin, which were detected at around 3.340, 10.203, 13.317, 16.681, 17.364, 21.909, and 26.472 min, respectively. The amounts of each chemical composition of *H. coronarium* extracts are shown in Table 2. Kojic acid, chlorogenic acid, EGCG, rutin, ellagic acid, and rosmarinic acid were found in different concentrations in both the leaf and the rhizome of *H. coronarium*. The leaf tended to contain a higher amount of the bioactive components than the rhizome. Chlorogenic acid and ellagic acid were identified as the predominant compounds in rhizome extracts obtained from both conventional maceration and PEF extraction methods. PEF was found to significantly enhance the content of chlorogenic acid, EGCG, rutin, ellagic acid, and rosmarinic acid, but specifically at 20 kV/cm (*p* < 0.05). On the other hand, the chemical profile of the leaf extract acquired through maceration varied from that obtained via PEF extraction. Kojic acid and EGCG predominated in the maceration-derived extract, while ellagic acid consistently stood out in all PEF extracts. In the leaf part, PEF extraction was not found to enhance the extraction of bioactive compounds, as the content of each compound, except for ellagic acid, was not comparable to those obtained from maceration. Nevertheless, increased electric field intensity in PEF correlated with heightened extraction efficiency, resulting in elevated content of all chemical compounds, particularly ellagic acid. The content of ellagic acid in the leaf extract obtained at 20 kV/cm during PEF reached 42.35 ± 1.64 mg/g extract, approximately ten times higher than that from maceration. Aside from ellagic acid, kojic acid was another compound found to be efficiently extracted by PEF. The probable explanation lies in its molecular size. Compounds with lower molecular weights (MWs), such as kojic acid (MW = 142.11 g/mol) and ellagic acid (MW = 302.197 g/mol), were found to be efficiently extracted with PEF assistance. In contrast, larger molecules such as rosmarinic acid (MW = 360.31 g/mol), chlorogenic acid (MW = 354.31 g/mol), EGCG (MW = 458.372 g/mol), and rutin (MW = 610.517 g/mol) extracted through the PEF method yielded comparatively lower amounts compared to conventional maceration methods. This could be attributed to the electroporation, which enables immediate passage of low-MW compounds across the cytoplasmic membrane of the plant cells, whereas larger molecules require an extended period for the pores to sufficiently enlarge, allowing their release [32]. PEF was suggested as a novel extraction method that significantly improved the efficiency of extracting bioactive compounds. Additionally, a shorter extraction period using PEF was more favorable in terms of energy efficiency compared to extending the extraction time through maceration [18].

Concerning the HPLC chromatograms of *H. coronarium* extracts, certain peaks remained unidentified. Additional analysis employing techniques such as gas chromatography-mass spectrometry (GC-MS) would be recommended for a more in-depth identification and characterization of the unknown peaks, which could elucidate the chemical compositions of the *H. coronarium* extracts. Despite using evaporation under vacuum conditions to remove the solvent from the PEF extraction, several terpenes and other volatile constituents could persist in the extracts. Notably, the ethanolic extract of *H. coronarium* rhizome was reported to contain benzene, 4-(1E)-1, 3-butadien-1-yl-1, 2-dimetho (20.78%), and 3-cyclohexen-1-ol, 4-methyl-1-(1-methyl-ethyl (20.78%) as major components [3].

### 2.3. Anti-Acne Activities of H. coronarium Extracts

**Figure 3:**
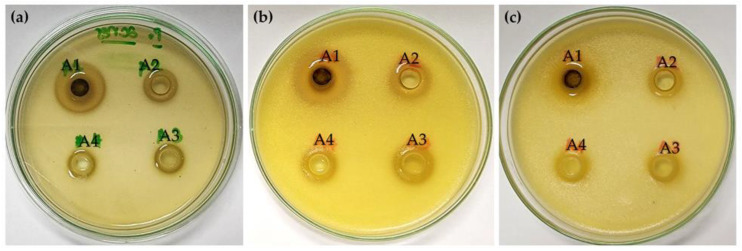
Photographs of a petri-dish used in the agar-well diffusion method for the PEF ethanolic extract derived from *H. coronarium* leaf at concentrations of 10 mg/mL (A1), 5 mg/mL (A2), 2.5 mg/mL (A3), and 1.3 mg/mL (A4) against *C. acnes* (**a**), *S. aureus* (**b**), and *M. luteus* (**c**). The diameter of the clear zone, indicating microbial inhibition, was measured.

The inhibitory activities against *C. acnes*, a microbe related to acne, are shown in Table 3. In the current investigation, all *H. coronarium* extracts were dissolved in 95% *v*/*v* ethanol; therefore, the anti-acne effect of 95% *v*/*v* ethanol was also tested to establish that the antimicrobial activity resulted from the extracts and not the solvents. Visible clear zones around wells treated with *H. coronarium* extract within an agar medium containing bacterial growth are shown in Figure 3a. *C. acnes* exhibited higher sensitivity to *H. coronarium* leaf extracts compared to the rhizome extracts. Although the inhibition zones of all *H. coronarium* leaf extracts were not different from each other, the MICs indicated that *C. acnes* exhibited higher sensitivity to the *H. coronarium* leaf extracts from PEF, particularly with the higher electrical field strength used in the PEF extraction. It was also highlighted that the MICs of the *H. coronarium* extract were lower than those of clindamycin, indicating a higher sensitivity of the bacterial strains to the extract. MIC and inhibition zone values could be used to categorize the antimicrobials into distinct groups, namely susceptible (S) and resistant (R). These were correlated with the probability of clinical outcomes, with S indicating a high probability of favorable treatment and R indicating a low probability [33]. However, resistance to clindamycin in *C. acnes* has been reported at an MIC of 0.25 μg/mL or higher, according to the European Committee on Antibiotic Susceptibility Testing (EUCAST), and at 8 μg/mL or higher, according to the Clinical & Laboratory Standards Institute (CLSI) [34]. Furthermore, *C. acnes* has been reported as the most commonly isolated anaerobe, with clindamycin resistance rates of 7.8% using CLSI breakpoints [35]. These would be the reason that clindamycin exhibited lower sensitivity against *C. acnes* compared to the *H. coronarium* extracts. Therefore, the *H. coronarium* leaf extract, particularly from 20 kV/cm-PEF extraction, was proposed as an antibacterial agent for acne. The substantial content of ellagic acid appeared to play a pivotal role in its anti-acne efficacy, demonstrated by a good correlation observed between the MIC value and the content of ellagic acid. Since the dissociation of protons from the hydroxyl groups of ellagic acid triggers hyper-acidification at the plasma membrane interface of microorganisms, an imbalance in the electrical charges across the membrane potentially causes cell death [36]. Furthermore, phenolic phytochemicals have recently been reported to exhibit dual redox behavior, displaying both pro-oxidant and antioxidant effects [37]. The pro-oxidant properties refer to the ability to induce oxidative stress [38], particularly through the generation of reactive oxygen species (ROS), which are highly antimicrobial against both Gram-positive and Gram-negative bacteria [39]. To verify the dual redox behavior of *H. coronarium* extracts, further evaluations of their antioxidant properties are suggested, as compounds with dual redox behavior are not only beneficial for antimicrobial purposes but also for their antioxidant effects.

### 2.4. Antimicrobial Activities Related to Deodorant Effects of H. coronarium Extracts

The antimicrobial activities related to deodorant effects of *H. coronarium* leaf and rhizome extracts are shown in Table 4 and Table 5. It was found that all *H. coronarium* extracts could inhibit the bacteria, which were known as the causes of body and foot odor. Antibacterial activity was observed in *S. aureus*, *B. subtilis*, *M. luteus*, *P. aeruginosa*, and *E. coli* as the clear zone with no bacterial growth, as shown in Figure 3. Although the inhibition zones of all *H. coronarium* extracts were not much different from each other, *H. coronarium* leaf extracts were found to be more potent than the rhizome regarding the lower MICs. Moreover, the *H. coronarium* leaf extract from 20 kV/cm-PEF extraction was the most effective in antibacterial activity against all bacteria tested in the current investigation, which included *S. aureus*, *B. subtilis*, *M. luteus*, *P. aeruginosa*, and *E. coli*. In summary, the *H. coronarium* leaf extract from 20 kV/cm-PEF extraction was proposed as an active component in deodorant products. The probable reason for this could be the presence of ellagic acid, which has well-documented antibacterial activities [40].

### 2.5. Irritation Properties of H. coronarium Extracts

**Figure 4:**
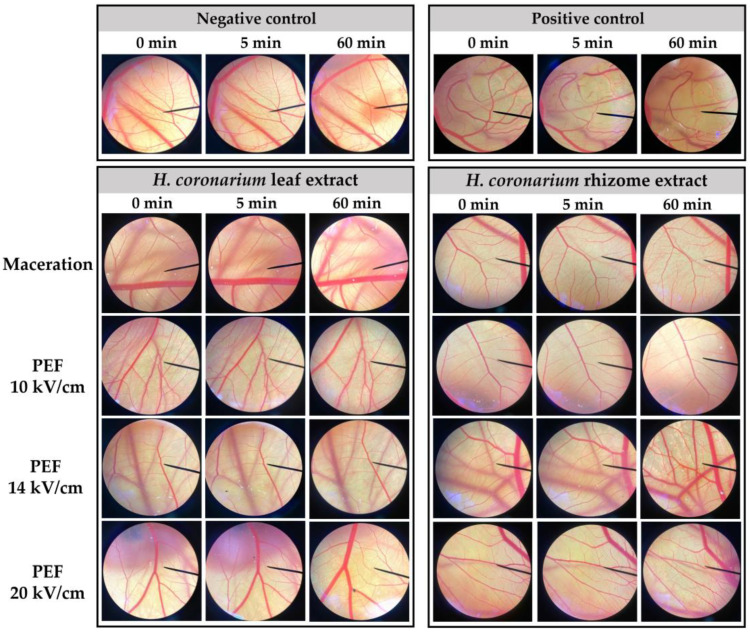
Irritation properties of *H. coronarium* extracts in HET-CAM test. PEF = pulse electric field extraction.

The hen’s egg test-chorioallantoic membrane (HET-CAM) is a reliable alternative method for determining the risk of chemical irritation [41,42]. It has been used to evaluate the irritation potential of various cosmetic ingredients, particularly those related to the eyes, or even facial care that has the possibility to irritate the eyes. The irritation properties of *H. coronarium* extracts were evaluated in the CAM, with results shown in Figure 4. It was noted that all samples were not found to be irritating since they induced an irritation sign and the IS was 0.0 ± 0.0, whereas the positive control induced severe irritation with an IS of 16.22 ± 0.4, as listed in Table 6. Therefore, the preliminary findings in the HET-CAM test suggested good tolerability of *H. coronarium* extracts since they induced no irritation.

## 3. Materials and Methods

### 3.1. Plant Materials

The leaves and rhizomes of *H. coronarium*, taken from Chulabhorn Royal Pharmaceutical Manufacturing Facility, Chon Buri, Thailand, underwent identification and authentication by Ms. Wannaree Charoensup, a botanist at the Herbarium, Department of Pharmaceutical Science, Faculty of Pharmacy, Chiang Mai University. The specimen number 0023314 was kept at the Herbarium of Faculty of Pharmacy, Chiang Mai University. Each sample of plant material was dried in a hot-air oven (Memmert, Schwabach, Germany) at a temperature of 45 °C for 72 h. The moisture contents of the dried powder of *H. coronarium* leaf and rhizomes were 6.8% and 7.5% on their dried basis, respectively. The dried *H. coronarium* was ground into fine powder and kept in a well-closed container protected from light and humidity until further use. The microscopic data of each dried powder were evaluated using a Nikon ECLIPSE E200 Microscope (Nikon Solutions Co., Ltd., Konan, Japan) connected with a Canon EOS750D camera (Canon Inc., Tochigi, Japan). The diagnostic pharmacognostic characters and cell components of each sample were examined with authentication by Ms. Wannaree Charoensup, a botanist at the Herbarium, Department of Pharmaceutical Science, Faculty of Pharmacy, Chiang Mai University. The microscopic data were photographed using a microscope with 400× lens magnifications.

### 3.2. Bacterial Strains

*Cutibacterium* (formerly *Propionibacterium*) acnes ATCC 6919, *Staphylococcus aureus* ATCC 25923, *Bacillus subtilis* TISTR 008, *Micrococcus luteus* TISTR 884, *Pseudomonas aeruginosa* ATCC 27853, and *E. coli* ATCC 25922 were obtained from the microbiological laboratory culture collection of the Faculty of Pharmacy at Chiang Mai University (CMU), Chiang Mai, Thailand. At 37 °C, the tested isolates were grown in Brain Heart Infusion (BHI) medium (HiMedia Laboratories, Mumbai, India).

### 3.3. Chemical Materials

Aluminum chloride, ampicillin, clindamycin hydrochloride, chlorogenic acid, epigallocatechin gallate (EGCG), ellagic acid, Folin–Ciocalteu reagent, formic acid, gallic acid, kojic acid, quercetin, rosmarinic acid, rutin trihydrate, sodium phosphate, disodium phosphate, sodium carbonate, potassium acetate, sodium chloride, sodium hydroxide, and sodium lauryl sulfate were analytical grade and purchased from Sigma-Aldrich Chemie GmbH, Taufkirchen, Germany. Acetonitrile was HPLC grade and purchased from Fisher Scientific (Pittsburgh, PA, USA).

### 3.4. Extraction Methodologies

#### 3.4.1. Conventional Solvent Extraction

Briefly, 100 g of dried powder of *H. coronarium* leaf or rhizome was macerated in 500 mL of 95% *v*/*v* ethanol for 24 h by shaking regularly at room temperature using an orbital shaker (Eppendorf, Hamburg, Germany) set at 150 rpm. The resulting extracts were filtered through Whatman No. 1 filter paper (Merck KGaA, Darmstadt, Germany) and the solvent pooled from three cycles of maceration was evaporated by a rotary evaporator (Buchi Evaporator, Heidolph, Germany) [43]. The extract was obtained and stored at 4 °C until the next usage. The yields of each extract were calculated using the following equation:

Yield (%) = A/B × 100,(1)

where A represents the weight of the extract and B represents the weight of the dried plant materials used in conventional solvent extraction.

#### 3.4.2. PEF Extraction

Briefly, 10 g of dried powder of *H. coronarium* leaf or rhizome was extracted via PEF (Faculty of Engineering, Rajamangala University of Technology Lanna, Chiang Mai, Thailand) using 95% *v*/*v* ethanol at a ratio of 1:20 in various PEF treatments, including 10, 14, and 20 kV/cm. The extraction process was adapted from a prior work conducted by Chaiyana et al. [44]. In brief, each extraction was performed at an ambient temperature for 5 min. The flyback circuit, which generates the high PEF voltage, is supplied with a 24 V, 200 W direct current switching power source [45]. After the extraction, the *H. coronarium* residue was removed by filtration through the qualitative Whatman No. 1 filter paper (Merck KGaA, Darmstadt, Germany). The extracting solvent from PEF extraction was removed by employing a rotary evaporator (Buchi Evaporator, Heidolph, Germany). The PEF extraction of each sample was performed in triplicate. The PEF extracts were obtained and stored at 4 °C until the next usage. The yields of each extract were calculated using the following equation:

Yield (%) = A/B × 100,(2)

where A represents the weight of the extract and B represents the weight of the dried plant materials used in the PEF extraction.

### 3.5. Chemical Composition Determination by High Performance Liquid Chromatography (HPLC)

The major content of the *H. coronarium* extracts was investigated via HPLC (Shimadzu Europe GmbH, Duisburg, Germany). The inertsil ODS-4 column (5 μm, 4.6 × 250 mm (UP), GL Sciences, Tokyo, Japan) was used as the stationary phase in combination with a guard column (5 μm, 4.6 × 10 mm). Each *H. coronarium* extract was dissolved in 95% *v*/*v* ethanol and filtered through a 0.45 μm nylon filter membrane (Hawach, Xi’an, China) before being injected with a volume of 20 µL into the HPLC system. A gradient elution with a flow rate of 1 mL/min using a mobile phase composed of a mixture of 100% acetonitrile (A) and 0.1% formic acid in DI water (B) was used as a mobile phase to elute the sample using the following gradient conditions: 0 min 90% A, 0–10 min 80% A, 10–25 min 60% A, 25–30 min 40% A, 30–35 min 40% A, 35–35.01 min 90% A, and 35.01–45 min 90% A. The mobile phase was filtered through a 0.45 μm nylon filter membrane (Hawach, Xi’an, China) and degassed in the sonication nylon filter membrane (Hawach, Xi’an, China) for 30 min prior to the injection. A diode array detector set at a wavelength of 280 nm was used to detect the chemical components of the *H. coronarium* extracts. Kojic acid, chlorogenic acid, EGCG, rutin trihydrate, ellagic acid, and rosmarinic acid were used as the reference standards in the HPLC analysis. All experiments were performed in triplicate.

### 3.6. Biological Activities Determination

#### 3.6.1. Bacteria Strain for Determination of Anti-Acne Activities

The anti-acne activities of *H. coronarium* extracts were investigated through their ability to inhibit *C. acnes* ATCC 6919 using the agar-well diffusion method [46]. Furthermore, the minimum inhibitory concentrations (MICs) were evaluated via the broth dilution method [47].

#### 3.6.2. Bacteria Strains for Determination of Antimicrobial Activities Related to Deodorant Effects

The deodorant effects of *H. coronarium* extracts were investigated through their ability to inhibit the microbial related to body odor, including *S. aureus* ATCC 25923, *B. subtilis* TISTR 008, *M. luteus* TISTR 884, *P. aeruginosa* ATCC 27853, and *E. coli* ATCC 25922, using the agar-well diffusion method [46]. Furthermore, the minimum inhibitory concentrations (MICs) were evaluated via the broth dilution method [47].

#### 3.6.3. Agar-Well Diffusion Method

The bacterial inoculum was diluted in PBS pH 7.4 to obtain turbidity visually comparable to the McFarland N° 0.5 standard (1 × 10^7^ CFU/mL). Each inoculum was spread over tryptic soy agar (TSA) plates. Prior to the investigation, an indicator strain was mixed with 1% *w*/*w* TSA to make a final concentration of 10^5^ CFU/mL. Subsequently, 10 mL of the TSA mixture was poured into a sterile petri-dish containing aluminum ring carriers having a 5 mm diameter (Faculty of Pharmacy, Chiangmai University, Chiang Mai, Thailand). After the TSA was set, wells were formed by removing the carriers. Following that, 100 µL of each extract with the concentration of 10 mg/mL was applied to the wells. After the incubation of 48 h at 37 °C [48], the inhibitory zones were observed by visual inspection and a Vernier Caliper (Mitutoyo, Kanagawa, Japan), and expressed in mm [46]. Clindamycin, ampicillin, and penicillin were used as reference antibiotics. All the experiments were carried out in triplicate.

#### 3.6.4. Broth Dilution Method

The minimum inhibitory concentrations (MICs) of each extract were determined by the broth dilution method [47]. Mueller–Hinton Broth (MHB) was placed in a 96-well plate (Corning Inc., Corning, NY, US). The extracts were then two-fold diluted in MHB at a ratio of 1:2 to 1:64, when the initial concentration of the extract was 10 mg/mL. The bacteria were added to test wells with a final concentration of 1 × 10^5^ CFU/mL and incubated at 37 °C for 24 h. The growth of bacteria was observed by visual inspection as follows: a clear solution indicated no growth, whereas a turbid cloudy solution indicated bacterial growth [49]. All experiments were carried out in triplicate.

### 3.7. Hen’s Egg-Chorioallantoic Membrane Test (HET-CAM Test)

The irritation of *H. coronarium* extracts was investigated via the HET-CAM test according to Chaiyana et al. [44]. This assay examined the irritation of test solution that led to difference adverse occurrence on the chorioallantoic membrane of the hen’s egg [47,50]. All eggs were incubated for 7 days in the hatching chamber set at 37.5 ± 0.5 °C and humidity of 55 ± 7% RH. The top of the eggshell was cut off with a rotating cutting blade (Ehwa Technologies Information Co., Ltd., Seoul, Republic of Korea). After that, the inner membrane was carefully removed using forceps to avoid breaking the vessel. Then, the sample solutions of 30 μL were exposed to chorioallantoic membrane (CAM). The irritation was recorded immediately after the exposures and observed for 5 min. The positive and negative controls of HET-CAM assay were aqueous solution of SLS (1% *w*/*v*) and normal saline solution (0.9% *w*/*v* NaCl), respectively. The irritation score (IS) was calculated and interpreted as following: 0.0–0.9 is no irritation, 1.0–4.9 is a mild irritation, 5.0–8.9 is a moderate irritation, and 9–21 is a severe irritation. After 60 min, the blood vessel networks were observed again under the microscope (Nikon, Tokyo, Japan).

### 3.8. Statistical Analysis

The resulting statistical significance was assessed using analysis of variance (ANOVA) followed by post-hoc test or unpaired *t*-test (SPSS 17.0 for Windows (SPSS Inc, Chicago, IL, USA)). The level of significant difference was set at * *p* < 0.05, ** *p* < 0.01, *** *p* < 0.001.

## 4. Conclusions

PEF was effectively used for the extraction of both the leaves and rhizomes of *H. coronarium.* As a consistent duration of 5 min was used for all PEF extractions, the PEF intensity was found to exhibit a significant effect on the extraction. Higher PEF intensity correlated with increased yields of *H. coronarium* ethanolic extracts. Notably, an intensity of 20 kV/cm yielded the highest content from both leaves and rhizomes, comparable to the extract yields obtained through a conventional maceration method involving three cycles of 24 h each, totaling three days. Despite equivalent extract yields from the leaf and rhizome, *H. coronarium* leaf extracts had a greater content of bioactive components indicated by HPLC in the current investigation. Remarkably, ellagic acid was a major component in *H. coronarium* leaf extracts, particularly those extracted by PEF at 20 kV/cm. Beside ellagic acid, various bioactive compounds were also detected in the *H. coronarium* extract, including kojic acid, chlorogenic acid, epigallocatechin gallate, rutin, and rosmarinic acid. The leaf part was found to be more potent than the rhizome in the inhibition against *B. subtilis* TISTR 008, *E. coli* ATCC 25922, *S. aureus* ATCC 25923, *M. luteus* TISTR 884, *P. aeruginosa* ATCC 27853, and *C. acnes* ATCC 6919. Interestingly, the *H. coronarium* extract from 20 kV-PEF/cm extraction displayed the most powerful inhibitory activity against all microorganisms, with the lowest minimum inhibitory concentrations compared to those of conventional extracts. In addition, all *H. coronarium* extracts were safe since they induced no irritation sign in the HET-CAM test. Therefore, PEF extraction is proposed as an environmentally friendly extraction process that could improve the extraction yield, the content of the biologically active compounds, and the antimicrobial effects of *H. coronarium* leaf, which are attractive for the cosmetic industry. In addition, PEF extraction is suggested as a sustainable extraction technique, supported by a significant reduction in extraction time and a remarkable decrease in solvent consumption.

## Figures and Tables

**Table 1:** Yield of *H. coronarium* extracts.

Extraction Methods	Yield (% *w*/*w* Based on Dried Plant Weight)	
	Rhizome	Leaf
Maceration	11.0 ± 2.7 ^a^	10.7 ± 1.0 ^a,b^
PEF 10 kV/cm	1.5 ± 0.6 ^d^	6.0 ± 0.1 ^b,c^
PEF 14 kV/cm	2.2 ± 0.2 ^c,d^	6.6 ± 0.4 ^b^
PEF 20 kV/cm	8.9 ± 1.0 ^a,b^	11.6 ± 3.2 ^a^

Note: PEF = pulse electric field extraction. Each extraction was repeated three times independently. The yields labeled with the same letter (a, b, c, and d) indicate no statistically significant difference between groups based on Tukey’s post hoc test at the significance level of *p* < 0.05. Groups with different letters are significantly different from each other (*p* < 0.05).

**Table 2:** Chemical compositions of *H. coronarium* extracts.

Extraction Methods	Amount (mg/g Dry Extract Weight)					
	Kojic Acid	Chlorogenic Acid	EGCG	Rutin	Ellagic Acid	Rosmarinic Acid
Rhizome						
Maceration	0.00 ± 0.00 ^a^	0.26 ± 0.01 ^b^	0.04 ± 0.00 ^b^	0.00 ± 0.00 ^b^	0.28 ± 0.07 ^b^	0.03 ± 0.00 ^b^
PEF 10 kV/cm	0.00 ± 0.00 ^a^	0.00 ± 0.00 ^d^	0.00 ± 0.00 ^c^	0.00 ± 0.00 ^b^	0.00 ± 0.00 ^c^	0.00 ± 0.00 ^b^
PEF 14 kV/cm	0.00 ± 0.00 ^a^	0.13 ± 0.00 ^c^	0.00 ± 0.00 ^c^	0.00 ± 0.00 ^b^	0.00 ± 0.00 ^c^	0.00 ± 0.00 ^b^
PEF 20 kV/cm	0.00 ± 0.00 ^a^	1.32 ± 0.02 ^a^	0.16 ± 0.01 ^a^	0.02 ± 0.00 ^a^	1.60 ± 0.05 ^a^	0.13 ± 0.02 ^a^
Leaf						
Maceration	16.90 ± 0.10 ^a^	1.84 ± 0.02 ^a^	15.28 ± 0.09 ^a^	4.06 ± 0.07 ^a^	4.27 ± 0.74 ^d^	7.22 ± 0.02 ^a^
PEF 10 kV/cm	1.72 ± 0.00 ^d^	0.04 ± 0.00 ^d^	1.21 ± 0.00 ^d^	0.17 ± 0.00 ^d^	8.24 ± 0.43 ^c^	0.53 ± 0.02 ^d^
PEF 14 kV/cm	4.28 ± 0.02 ^c^	0.36 ± 0.00 ^c^	3.30 ± 0.02 ^c^	0.93 ± 0.01 ^c^	15.35 ± 0.46 ^b^	0.86 ± 0.00 ^c^
PEF 20 kV/cm	10.84 ± 0.07 ^b^	0.79 ± 0.03 ^b^	6.97 ± 0.02 ^b^	1.58 ± 0.03 ^b^	42.35 ± 1.64 ^a^	2.85 ± 0.02 ^b^

Note: PEF = pulse electric field extraction; EGCG = epigallocatechin gallate. The amount of each chemical compound is reported in terms of percentage content, which refers to the dry extract weight. The values labeled with the same letter (a, b, c, and d) indicate no statistically significant difference between groups based on Tukey’s post hoc test at the significance level of *p* < 0.05. Groups with different letters are significantly different from each other (*p* < 0.05).

**Table 3:** Antibacterial activity related to acne of *H. coronarium* extracts.

Samples	*C. acnes* Inhibition Zone (mm)		MIC (µg/mL)	
	Rhizome	Leaf	Rhizome	Leaf
Clindamycin 10 µg/mL	40.2 ± 0.2 ^a^		≥4.9	
Penicillin G 50 µg/mL	16.1 ± 0.4 ^c^		≥0.8	
Ampicillin 50 µg/mL	19.8 ± 0.6 ^b^		≥0.4	
95% *v*/*v* Ethanol	8.5 ± 0.1 ^e^		ND	
*H. coronarium* extracts				
Maceration	9.8 ± 0.0 ^d^	10.2 ± 0.0 ^d^	≥1.3	≥0.6
PEF 10 kV/cm	9.1 ± 0.1 ^e^	9.8 ± 0.0 ^d^	≥1.3	≥5.0
PEF 14 kV/cm	8.2 ± 0.0 ^e^	8.4 ± 0.1 ^d^	≥1.3	≥1.3
PEF 20 kV/cm	9.7 ± 0.1 ^d^	9.9 ± 0.1 ^d^	≥1.3	≥0.6

Note: MIC = minimum inhibitory concentration; PEF = pulse electric field extraction; ND = not determined. The diameter size of the disc was 5 mm. The values labeled with the same letter (a, b, c, d, and e) indicate no statistically significant difference between groups based on Tukey’s post hoc test at the significance level of *p* < 0.05. Groups with different letters are significantly different from each other (*p* < 0.05).

**Table 4:** Antibacterial activities related to deodorant effects of *H. coronarium* extracts.

Samples	Inhibition Zone (mm)				
	*S. aureus*	*B. subtilis*	*M. luteus*	*P. aeruginosa*	*E. coli*
Clindamycin 10 µg/mL	44.3 ± 0.4 ^a^	40.2 ± 0.4 ^a^	30.6 ± 0.5 ^a^	29.2 ± 0.3 ^a^	28.5 ± 0.3 ^a^
Penicillin G 50 µg/mL	30.0 ± 0.4 ^c^	16.4 ± 0.2 ^c^	25.6 ± 0.8 ^b^	22.5 ± 0.2 ^b^	22.3 ± 0.2 ^b^
Ampicillin 50 µg/mL	35.5 ± 0.3 ^b^	19.0 ± 0.2 ^b^	15.3 ± 0.4 ^c^	14.1 ± 0.2 ^c^	14.7 ± 0.1 ^c^
95% *v*/*v* Ethanol	7.8 ± 0.1 ^e^	8.5 ± 0.1 ^e^	9.0 ± 0.1 ^e^	9.5 ± 0.1	9.7 ± 0.1 ^d^
Rhizome					
Maceration	9.5 ± 0.1 ^d^	11.2 ± 0.0 ^d^	10.8 ± 0.0 ^d^	10.8 ± 0.0 ^d^	9.5 ± 0.0 ^d^
PEF 10 kV/cm	8.2 ± 0.0 ^e^	8.8 ± 0.1 ^e^	9.8 ± 0.0 ^e^	9.8 ± 0.0 ^e^	9.1 ± 0.0 ^d^
PEF 14 kV/cm	8.1 ± 0.0 ^e^	9.4 ± 0.1 ^e^	9.2 ± 0.1 ^e^	9.2 ± 0.1 ^e^	8.1 ± 0.1 ^e^
PEF 20 kV/cm	8.3 ± 0.0 ^e^	9.4 ± 0.0 ^e^	9.8 ± 0.0 ^e^	9.8 ± 0.0 ^e^	9.2 ± 0.1 ^d^
Leaf					
Maceration	10.1 ± 0.2 ^d^	12.7 ± 0.0 ^d^	12.3 ± 0.1 ^d^	11.8 ± 0.1 ^d^	9.7 ± 0.1 ^d^
PEF 10 kV/cm	9.5 ± 0.1 ^d^	9.4 ± 0.0 ^e^	11.2 ± 0.1 ^d^	11.2 ± 0.1 ^d^	9.7 ± 0.0 ^d^
PEF 14 kV/cm	8.2 ± 0.0 ^e^	10.0 ± 0.1 ^d^	9.2 ± 0.1 ^e^	9.2 ± 0.1 ^e^	8.1 ± 0.1 ^e^
PEF 20 kV/cm	9.2 ± 0.0 ^d^	10.7 ± 0.1 ^d^	10.8 ± 0.0 ^d^	10.8 ± 0.0 ^d^	9.1 ± 0.1 ^d^

Note: PEF = pulse electric field extraction. The diameter size of the disc was 5 mm. The values labeled with the same letter (a–e) indicate no statistically significant difference between groups based on Tukey’s post hoc test at the significance level of *p* < 0.05. Groups with different letters are significantly different from each other (*p* < 0.05).

**Table 5:** Minimum inhibitory concentration on bacterial related to deodorant effects of *H. coronarium* extracts.

Samples	MIC (µg/mL)				
	*S. aureus*	*B. subtilis*	*M. luteus*	*P. aeruginosa*	*E. coli*
Clindamycin	≥4.9	≥4.9	≥4.9	≥4.9	≥9.8
Penicillin G	≥0.4	≥0.4	≥0.8	≥0.4	≥0.4
Ampicillin	≥0.4	≥0.4	≥0.4	≥0.4	≥0.8
Rhizome					
Maceration	≥1.3	≥1.3	≥1.3	≥2.5	≥2.5
PEF 10 kV/cm	≥5.0	≥5.0	≥2.5	≥2.5	≥2.5
PEF 14 kV/cm	≥1.3	≥1.3	≥1.3	≥5.0	≥5.0
PEF 20 kV/cm	≥2.5	≥1.3	≥1.3	≥2.5	≥2.5
Leaf					
Maceration	≥1.3	≥0.6	≥0.6	≥0.6	≥1.3
PEF 10 kV/cm	≥1.3	≥1.3	≥1.3	≥0.6	≥1.3
PEF 14 kV/cm	≥5.0	≥1.3	≥1.3	≥5.0	≥5.0
PEF 20 kV/cm	≥1.3	≥0.6	≥0.6	≥0.6	≥1.3

Note: MIC = minimum inhibitory concentration; PEF = pulse electric field extraction. The diameter size of the disc was 5 mm.

**Table 6:** Irritant score and irritancy classification in HET-CAM test.

Substance	Irritation Score (IS)	Irritancy Classification
Positive control (SLS)	16.22 ± 0.4 ^a^	Severe irritation
Negative control (NSS)	0.0 ± 0.0 ^b^	No irritation
Rhizome		
Maceration	0.0 ± 0.0 ^b^	No irritation
PEF 10 kV/cm	0.0 ± 0.0 ^b^	No irritation
PEF 14 kV/cm	0.0 ± 0.0 ^b^	No irritation
PEF 20 kV/cm	0.0 ± 0.0 ^b^	No irritation
Leaf		
Maceration	0.0 ± 0.0 ^b^	No irritation
PEF 10 kV/cm	0.0 ± 0.0 ^b^	No irritation
PEF 14 kV/cm	0.0 ± 0.0 ^b^	No irritation
PEF 20 kV/cm	0.0 ± 0.0 ^b^	No irritation

Note: SLS = 1% *w*/*v* Sodium lauryl sulfate aqueous solution; NSS = normal saline solution, which was 0.9% *w*/*v* NaCl aqueous solution. PEF = pulse electric field extraction; The values labeled with the same letter (a and b) indicate no statistically significant difference between groups based on Tukey’s post hoc test at the significance level of *p* < 0.05. Groups with different letters are significantly different from each other (*p* < 0.05).

## Data Availability

Data available on request.
